# Correlations between clinical insomnia and psychophysiological factors in postoperative patients discharged from the ICU: A cross‐sectional study

**DOI:** 10.1002/pcn5.70081

**Published:** 2025-03-20

**Authors:** Nobuo Sato, Kentaro Matsui, Masako Arakida, Rie Akaho, Katsuji Nishimura, Takeshi Nomura

**Affiliations:** ^1^ Department of Anesthesiology St. Marianna University School of Medicine Kanagawa Japan; ^2^ Department of Intensive Care Medicine Tokyo Women's Medical University Tokyo Japan; ^3^ Department of Clinical Laboratory National Center Hospital, National Center of Neurology and Psychiatry Tokyo Japan; ^4^ Department of Psychiatry Tokyo Women's Medical University Tokyo Japan; ^5^ Department of Sleep‐Wake Disorders National Institute of Mental Health, National Center of Neurology and Psychiatry Tokyo Japan; ^6^ Department of Nursing Tokyo Women's Medical University Hospital Tokyo Japan

**Keywords:** critical illness, depression, fatigue, insomnia, intensive care unit, post‐intensive care syndrome, post‐traumatic stress disorder, sleep

## Abstract

**Aim:**

Post‐intensive care unit (ICU) patients often experience physical or mental dysfunction. This study aims to investigate the relationship between sleep disturbances and mental and physical problems in patients discharged from the ICU to hospital wards, using both subjective and objective sleep measures.

**Methods:**

Patients admitted to the ICU for at least 72 h between November 2021 and June 2022 were included in the study. On the seventh day after ICU discharge, we conducted an objective sleep assessment using an electroencephalogram‐based mobile sleep‐monitoring device. Patients self‐administered severity ratings for fatigue, depression, and post‐traumatic stress (PTS) symptoms. Insomnia symptoms were assessed using the insomnia severity index (ISI) before and after ICU admission.

**Results:**

Thirty‐five patients (median age 73) were included in the study. Higher ISI scores at ward were significantly correlated with higher levels of fatigue (*r* = 0.463, *P* = 0.005), depression (*r* = 0.343, *P* = 0.044), and PTS symptoms (*r* = 0.477, *P* = 0.004). Among the objective sleep measures, reduced N3 sleep (*r* = −0.480, *P* = 0.004) was significantly correlated with more severe PTS symptoms. However, no statistically significant correlations were found between objective sleep indicators and either fatigue or depression.

**Conclusion:**

The observed reduction in N3 sleep and its association with PTS symptoms in this study might have reflected the physical and psychological stress experienced during intensive care. Subjective insomnia severity, which was related to fatigue, depression, and PTS symptoms in the ward, could be an important intervention target after ICU discharge.

## INTRODUCTION

Intensive care units (ICUs) play a crucial role in modern healthcare, significantly improving survival rates for critically ill patients.^1^, ^2^ However, this success has also presented a new challenge: many ICU survivors experienced long‐term sequelae collectively known as post‐intensive care syndrome (PICS), including persistent cognitive, motor, and respiratory dysfunctions that persisted after hospital discharge.^3^, ^4^, ^5^ One significant aspect of post‐ICU recovery is sleep disruption. Admission to the ICU can lead to sleep disruption, caused by environmental factors such as noise and light, medical factors including pain, respiratory distress, and sleep‐disrupting medications, and psychological factors such as stress and anxiety.^6^, ^7^, ^8^, ^9^ Recently, sleep disturbances in the ICU have been recognized as a potential cause of PICS,^10^ with evidence indicating that subjective sleep disturbances, particularly insomnia, may affect both the onset and outcomes of PICS.^11^

In evaluating sleep states, objective assessments such as polysomnography (PSG), based on electroencephalography (EEG) data,^12^, ^13^ were conducted, and the relationship between these objective sleep indicators and health outcomes was established.^14^ Assessments using PSG indicated that over 50% of ICU patients, including all those on mechanical ventilation, experience sleep issues.^15^ Our previous study, which involved EEG measurements of sleep in ICU patients, revealed that a majority of patients exhibited objective sleep fragmentation.^16^ This sleep fragmentation can persist even after leaving the ICU, as studies have reported that critically ill patients continue to experience reduced sleep efficiency and shortened sleep duration even after transitioning from the ICU to a step‐down unit.^6^, ^17^ However, the correlation between insomnia symptoms in general wards and objective sleep indicators remains unexplored. Moreover, the association between post‐ICU sleep problems and physical/mental functional impairments has not been thoroughly investigated.

The recovery process of physical and mental functions continues after patients are discharged from the ICU. Regular assessment of these functions in the ward and the implementation of appropriate interventions are crucial for optimal recovery during this period. These assessments can support the early detection and management of PICS; identifying risk factors and preventive strategies for PICS could lead to the development of new interventions to improve the long‐term prognosis of critically ill patients. Therefore, understanding the factors that influence post‐ICU recovery, particularly sleep, is essential. The primary aim of this study was to examine the relationship between post‐ICU discharge health outcomes—including daily activities, fatigue, depression, and post‐traumatic stress (PTS)—and both subjective and objective sleep indicators. Additionally, we explored differences in objective sleep indicators based on the presence or absence of clinical insomnia in these patients, complementing our primary objective.

## MATERIALS AND METHODS

### Study setting and sample

The study was conducted from November 2021 to June 2022 at Tokyo Women's Medical University Hospital, a 1200‐bed tertiary teaching hospital in Tokyo, Japan. Patients aged 20 years or older who had undergone surgery and were subsequently admitted to the ICU for at least 72 h were eligible for this study. Exclusion criteria included a history of brain dysfunction, psychiatric disorders, dementia, alcohol or drug abuse, or post‐cardiopulmonary arrest. Informed consent was obtained from all patients in the ICU after confirming their full consciousness and decision‐making capacity.

### Data collected upon consent

During the consent acquisition process, cognitive function was evaluated by trained researchers (N.S. or M.A.) using the Mini‐Mental State Examination (MMSE). The MMSE is a widely used tool for quantifying cognitive function, particularly in elderly populations, with lower scores indicating declining cognitive abilities.^18^ To assess the severity of insomnia before the surgery, participants were asked to recall and report their insomnia symptoms prior to ICU admission using the Insomnia Severity Index (ISI).^19^, ^20^ This widely used tool provides a comprehensive and reliable measure of insomnia severity and is also highly feasible given its quick and simple administration. The ISI is a self‐administered questionnaire that includes seven items: the severity of sleep onset, sleep maintenance, and early morning waking problems, satisfaction with current sleep pattern, interference with daily functioning, noticeability of impairment attributed to the sleep problem, and degree of distress or concern caused by the sleep disturbance. Each item is rated on a scale from 0 to 4, representing a range from “no problem” to “very severe problem.” The total scores are categorized as follows: absence of insomnia (0–7), subthreshold insomnia (8–14), moderate insomnia (15–21), and severe insomnia (22–28). We categorized moderate or severe insomnia (ISI ≥ 15) as the clinical insomnia group and otherwise (ISI < 15) as the subthreshold or non‐insomnia group.^21^

### Data collection during sleep‐monitoring

After discharge from the ICU, subjective assessments of sleep, physical, and mental symptoms were conducted in the general ward. These subjective assessments were conducted prior to the sleep‐monitoring with the ZA‐X (an EEG‐based mobile sleep‐monitoring device, detailed in the following section) on the same day. The ISI was re‐administered to assess changes in insomnia severity after ICU discharge. Additionally, the Barthel Index, the Fatigue Severity Scale (FSS), the Overall Depression Severity and Impairment Scale (ODSIS), and the Impact of Event Scale‐Revised (IES‐R) were evaluated.

The Barthel Index is a severity scale used to assess functional independence and activities of daily living (ADL) in individuals with various health conditions, particularly those with physical disabilities or impairments. It measures the individual's ability to perform 10 basic activities, including feeding, bathing, grooming, dressing, bowel and bladder control, toileting, transferring, mobility, and stair climbing. The total score is expressed from 0 to 100 points, with higher scores indicating greater independence and lower scores indicating greater dependence.^22^ The FSS is a widely used instrument for quantifying fatigue levels in individuals. The FSS consists of nine items that assess the impact of fatigue on daily activities, motivation, and the ability to carry out tasks. Each item is rated on a seven‐point Likert scale (1 = strongly disagree, 7 = strongly agree). The total score is calculated by averaging the scores of all items, with higher scores indicating greater fatigue severity.^23^ The ODSIS is a five‐item rating scale that assesses severity and functional impairment due to depressive symptoms, including frequency and intensity of depression and interference with work, school, social life, and relationships. It also assesses disability due to depression‐related loss of interest and difficulty participating in activities.^24^ All of these questions are rated on a Likert scale from 0 to 4 and refer to a “past week” time frame, with higher scores indicating greater severity and impairment related to depression. The IES‐R is a widely used tool for assessing the severity of PTS symptoms. It is designed to measure the subjective distress experienced by individuals following a traumatic event. The IES‐R consists of 22 items that assess the frequency of intrusive thoughts, avoidance behaviors, and hyperarousal symptoms related to the traumatic event. Each item is rated on a five‐point Likert scale (0 = not at all, 4 = extremely). The total score is calculated by summing the scores of all items, with higher scores indicating greater severity of PTS symptoms.^23^

### Objective sleep assessment using EEG‐based mobile sleep‐monitoring device

The ZA‐X is a two‐channel mobile sleep‐monitoring device developed by Proassist (Osaka, Japan) that can determine sleep stages similar to PSG, providing a reliable method for objective sleep assessment. Both ZA‐X recording and analysis methods are described in detail elsewhere.^25^ Briefly, the electrode for ZA‐X's channel 1 was placed 1 cm above and slightly lateral to the outer canthus of the left eye and right mastoid. Two additional disposable electrodes for channel 2 were placed 1 cm below and slightly lateral to the outer canthus of the right eye as well as above the chin. Data collected from the ZA‐X were transferred to a cloud service (Sleep Diver, Proassist), where spectral analysis of the EEG data was performed every 30‐s epoch. The data were analyzed in five stages: wakefulness, stage 1 (N1), stage 2 (N2), stage 3 (N3), and rapid eye movement sleep (REM). Stage information was provided along with time stamps, and EEG traces were available for download. This sleep stage analysis service was approved for medical device certification (301AGBZX00091000) in Japan. In this study, ZA‐X recordings were made from 7:00 pm on the seventh day following ICU discharge to 7:00 am, with adjustments to start and finish times permitted based on patient needs or clinical care. The total sleep time (TST) was calculated as the sum of N1, N2, N3, and REM stages from the 12 h recordings. Additional parameters included sleep latency (SL), REM sleep latency (REM SL), and wake time after sleep onset (WASO). The morning following the sleep‐monitoring, patients reported their subjective sleep duration from the previous night. The sleep misperception index, calculated by subtracting subjective sleep duration from objective sleep duration (i.e., TST), was then evaluated, potentially indicating health‐related issues.^26^, ^27^

### Data from medical records

Demographic and clinical data including age, sex, body mass index, type of surgery, period of artificial respiration, medications used, length of ICU stay, and length of hospitalization were collected from the patients' electronic medical records. The Acute Physiology and Chronic Health Evaluation (APACHE) II scores, which assess patient severity in the ICU, were calculated based on data including age, physiological parameters (e.g., blood pressure, heart rate, and respiratory rate), and chronic health conditions (e.g., pre‐existing conditions, and immune status) within 24 h of ICU admission and graded on a scale of 0 to 71, with higher scores indicating greater severity. The delirium symptoms were assessed using standardized tools: the Intensive Care Delirium Screening Checklist (ICDSC) in the ICU, with a score of 4 or higher indicating delirium,^28^, ^29^ and the Delirium Rating Scale‒Revised‒98 (DRS‐R‐98) in the general ward, with a score of 10 or higher indicating delirium.^30^, ^31^ The ICDSC was administered twice a day during the ICU stay, and the DRS‐R‐98 was administered in the general ward for at least 5 days; the number of days exceeding the respective cutoffs shown above was counted as the number of days with delirium.

### Statistical analyses

Participants were stratified into two groups based on their ISI score at the ward: a clinical insomnia group (ISI ≥ 15) and a subthreshold/non‐insomnia group (ISI < 15). Objective sleep parameters by ZA‐X (TST, SL, REM SL, WASO, N1, N2, and N3), subjective sleep duration, and sleep misperception index were compared between the two groups using Mann–Whitney U test. Spearman's correlation analysis was used to examine the relationships between (1) the sleep parameters (including ZA‐X measures, subjective sleep duration, and ISI score at the ward) and (2) the Barthel Index, FSS, ODSIS, and IES‐R evaluated on the day of sleep‐monitoring. Statistical analyses were performed using SPSS Statistics version 26 J (SPSS Japan, Inc). Statistical significance was set at *P* < 0.05.

## RESULTS

Of the patients admitted to the ICU during the study period, 63 met the inclusion criteria. Thirty‐eight patients were excluded due to discharge within 7 days of ICU discharge (*n* = 2), lack of 12‐hour sleep measurement (*n* = 23), or electrode disconnection during ZA‐X measurement (*n* = 3). Finally, 35 patients (23 male and 12 female) were included in this study. The subjects had a median age of 73 years. The median ISI before ICU admission was 5 points. After discharge from the ICU, the median scores for ISI, Barthel Index, FSS, ODSIS, and IES‐R on the ward were 12, 95, 3.4, 0, and 9, respectively. Seventeen out of 35 patients (48.6%) developed delirium; however, delirium had resolved in all affected patients at least 7 days prior to EEG recordings. Ten patients (28.6%) took a hypnotic drug on the day of their sleep EEG measurement, with most receiving either melatonin receptor agonists or orexin receptor antagonists, except for one patient who took clonazepam. Other demographic and clinical characteristics, including BMI, MMSE scores, APACHE2 scores, types of surgery, and length of stay, are summarized in Table 1.

**Table 1:** Demographic data and characteristics of the subjects (*n* = 35).

Characteristics	
Male, *n* (%)	23 (65.7)
Age, median [range], year	73 [29–89]
Body mass index, median [range], kg/m^2^	23.2 [17.3–31.1]
MMSE, median [range], point	29 [21–30]
Regular use of sleeping pills pre‐ICU, *n* (%)	3 (8.6)
ISI before ICU admission, median [range], point	5 [0–18]
APACHE II, median [range], point	17 [10–25]
Admission reason	
Post cardiothoracicvascular surgery, *n* (%)	30 (85.7)
Post gastrointestinal surgery, *n* (%)	4 (11.4)
Post head and neck surgery, *n* (%)	1 (2.9)
Artificial respiration period, median [range], day	2 [1–20]
Days of ICU stay, median [range], day	6 [5–24]
Days of hospitalization, median [range], day	31 [18–125]
Postoperative delirium, *n* (%)	17 (48.6)
Days with delirium, median [range], day	2 [1–10]
Measurements at the ward	
Use of sleeping pills at the ward, *n* (%)	10 (28.6)
Benzodiazepine, *n*	1
Melatonin receptor agonist, *n*	6
Orexin receptor antagonist, *n*	4
ISI at ward, median [range], point	12 [0–25]
Barthel Index, median [range], point	95 [0–100]
FSS, median [range], point	3.4 [1.0–7.0]
ODSIS, median [range], point	0 [0–15]
IES‐R, median [range], point	9 [0–64]

Abbreviations: APACHE II, Acute Physiology and Chronic Health Evaluation II; BMI, body mass index; FSS, Fatigue Severity Scale; IES‐R, Impact of Event Scale‐Revised; ISI, Insomnia Severity Index; MMSE, Mini‐Mental State Examination; ODSIS, Overall Depression Severity and Interference Scale.

The descriptive statistics for the 35 patients who underwent ZA‐X measurements are presented in Table 2. The duration of ZA‐X recording had a median of 699 min, with a range from 532 to 998 min. The median TST was 358.5 min, ranging from 169 to 570.5 min. Subjective sleep duration was reported with a median of 300 min, varying from 60 to 540 min (data on subjective sleep duration was missing for one participant in the clinical insomnia group). The median sleep misperception index was −30.5 min, with a broad range from −450.5 to 191 min. No significant differences were found between the clinical insomnia group and the subthreshold/non‐insomnia group in any ZA‐X sleep parameters or self‐reported sleep duration. However, the effect sizes (*r*) for TST, SL, N2, and subjective sleep duration exceeded 0.2, indicating a potential, albeit small, difference between the groups in these specific parameters (Table 2).

**Table 2:** Sleep parameters of patients assessed via mobile sleep‐monitoring device (*n* = 35).

Sleep parameter	All (*n* = 35)	Clinical insomnia group (*n* = 16)^a^	Subthreshold or non‐insomnia group (*n* = 19)^b^	*P*	*r* c
TST, median [range], min	358.5 [169–570.5]	329.25 [169–473]	366.5 [272.5–570.5]	0.182	0.227
WASO, median [range], min	140 [49.5–460]	163.5 [49.5–292.5]	132.5 [57.5–460]	0.589	0.095
SL, median [range], min	119.5 [7.5–253.5]	150.75 [26.5–199]	118 [7.5–253.5]	0.217	0.210
REM SL, median [range], min	121.5 [38.5–444.5]	135.75 [61–444.5]	118.5 [38.5–232.5]	0.385	0.151
N1, median [range], min	33 [11–104]	36.25 [14.5–74]	27.5 [11–104]	0.781	0.050
N2, median [range], min	176.5 [79.5–307.5]	141.5 [79.5–275.5]	182.5 [104–307.5]	0.133	0.255
N3, median [range], min	57.5 [9–142]	52.75 [9–135.5]	61 [10.5–142]	0.441	0.134
REM, median [range], min	75.5 [10–150]	72.5 [10–150]	75.5 [24–147.5]	0.441	0.134
Subjective sleep duration, median [range], min^d^	300 [60–540]	270 [60–420]	360 [60–540]	0.198	0.224
Sleep misperception index, median [range], min^d^^,^ e	30.5 [−191–450.5]	54.25 [−191–263]	17.5 [−174–450.5]	0.932	0.018

Abbreviations: REM, rapid eye movement sleep; SL, sleep latency; TIB, time in bed; TST, total sleep time; WASO, wake time after sleep onset.

^a^ Insomnia severity index (ISI) ≥ 15.

^b^ ISI < 15.

^c^ Calculated as the statistic divided by the square root of the number of subjects.

^d^ Listwise deletion was applied for one case with missing subjective sleep duration data within the clinical insomnia group.

^e^ Computed by subtracting subjective sleep duration from objective sleep duration (i.e. TST).

Table 3 presents the bivariate correlations between sleep parameters (including ZA‐X measures, subjective sleep duration, and ISI score) and outcomes (ADL, fatigue, depressive symptoms, and PTS symptoms) assessed at the ward. Shorter SL was correlated with lower Barthel Index scores (*r* = 0.382, *P* = 0.024). Reduced N3 (*r* = −0.480, *P* = 0.004) and a more negative sleep misperception index (*r* = −0.361, *P* = 0.036) were also significantly correlated with higher IES‐R scores. Higher ISI scores at ward were significantly correlated with higher FSS (*r* = 0.463, *P* = 0.005), ODSIS (*r* = 0.343, *P* = 0.044), and IES‐R scores (*r* = 0.477, *P* = 0.004). Other correlations between sleep indicators and the Barthel Index, FSS, ODSIS, or IES‐R were not statistically significant.

**Table 3:** Bivariate correlation between measurements at the ward and sleep parameters in patients assessed via mobile sleep‐monitoring device (*n* = 35).

	Barthel Index		FSS		ODSIS		IES‐R	
	*r*	*P*	*r*	*P*	*r*	*P*	*r*	*P*
TST	−0.270	0.117	0.032	0.856	0.149	0.392	−0.149	0.393
WASO	−0.135	0.438	0.052	0.765	−0.155	0.375	−0.077	0.662
SL	0.382*	0.024	−0.096	0.584	0.256	0.138	0.228	0.188
REM SL	−0.030	0.862	0.05	0.773	0.012	0.946	−0.108	0.537
N1	−0.212	0.221	−0.024	0.889	0.045	0.798	−0.155	0.375
N2	−0.065	0.709	−0.115	0.512	0.117	0.503	0.045	0.796
N3	−0.092	0.597	−0.055	0.753	−0.181	0.298	−0.480**	0.004
REM	−0.200	0.249	0.126	0.470	0.030	0.862	0.047	0.789
Subjective sleep duration^a^	−0.078	0.661	−0.193	0.275	0.268	0.126	0.254	0.147
Sleep misperception index^a^^,^ b	−0.073	0.681	0.125	0.483	−0.225	0.200	−0.361*	0.036
ISI (at the ward)	0.051	0.769	0.463**	0.005	0.343*	0.044	0.477**	0.004

Abbreviations: FSS, Fatigue Severity Scale; IES‐R, Impact of Event Scale‐Revised; ODSIS, Overall Depression Severity and Interference Scale; REM, rapid eye movement sleep; SL, sleep latency; TIB, time in bed; TST, total sleep time; WASO, wake time after sleep onset.

^a^ Listwise deletion was applied for one case with missing subjective sleep duration data.

^b^ Computed by subtracting subjective sleep duration from objective sleep duration (i.e. TST).

** *P* < 0.01

* *P* < 0.05.

## DISCUSSION

We comprehensively assessed physical and mental health outcomes, as well as sleep quality, in patients who underwent surgery and were admitted to the ICU before being discharged to a ward. Our findings revealed significant correlations between subjective insomnia severity and fatigue, depressive symptoms, and post‐traumatic stress (PTS) symptoms. Among the objective sleep parameters, reduced N3 sleep was correlated with higher PTS symptom scores. These results suggest that both subjective and objective sleep disturbances may have implications for mental health outcomes in this patient group, highlighting the importance of sleep quality in post‐ICU recovery.

Our study uniquely incorporated both subjective and objective sleep measures, allowing for a more comprehensive understanding of post‐ICU sleep patterns. Subjective evaluations assess the patients' perceived quality and satisfaction of sleep, while objective measurements like PSG provide highly reproducible indices that reflect the physiological basis of sleep, potentially making them more important for prognostic indicators than subjective assessments.^32^ In the present study, sleep was evaluated using both objective measures through an EEG‐based mobile sleep‐monitoring device and subjective evaluations via surveys. Comparing sleep parameters between clinical insomnia groups and subthreshold or non‐insomnia groups revealed no statistically significant differences in subjective sleep duration or objective sleep parameters. However, effect size analysis suggested that the clinical insomnia group tended to have longer sleep latency, shorter TST, shorter stage N2 sleep, and shorter subjective sleep duration, which are consistent with characteristics of insomnia in the elderly.^33^ In chronic insomnia, subjective sleep duration is typically significantly shorter than objective sleep duration.^34^ However, in this study, the discrepancy between objective and subjective sleep durations was minimal across groups. This minimal misperception of sleep states may suggest that acute sleep disturbance after intensive care differs from typical chronic insomnia disorders.

Our correlation analysis revealed an unexpected relationship between physical activity levels and sleep patterns; individuals with higher physical activity levels, as measured by the Barthel Index, experienced longer objectively measured sleep latencies. This finding contrasts with previous studies suggesting a correlation between lower physical activity levels and poorer sleep quality.^35^, ^36^ One possible explanation for this finding is the long sleep measurement period (7 pm to 7 am) used in this study. Considering that the average TST for the participants was about 6 h, it appears that those with lower physical activity levels might have been falling asleep too early. This is generally considered detrimental in insomnia treatment and is typically advised against.^37^ Interventions that reduce time spent in bed at night may be effective in treating insomnia symptoms, even in post‐intensive care patients. While this study suggests that temporary reductions in physical functioning post‐surgery may not significantly impact insomnia symptoms, variations in physical activity levels could still influence sleep latency, highlighting the complexity of post‐ICU recovery.

Numerous studies have examined the relationship between insomnia and both depression^38^, ^39^, ^40^ and fatigue^41^, ^42^ in the general population. Consistent with this, our results showed that both depression and fatigue were significantly correlated with subjective insomnia symptoms. However, neither depression nor fatigue were correlated with objective sleep measurements in this study. These findings are also supported by the previous studies, which have shown that the relationship between depression, fatigue, and subjective indicators of sleep is clearer than the relationship with objective indicators of sleep.^42^, ^43^, ^44^ On the other hand, other studies using continuous actigraphy measurements over periods ranging from 2 to 30 days showed pronounced associations with levels of fatigue^45^, ^46^ and depression.^47^ As this study only evaluated data from a single night, future studies may benefit from using continuous sleep data to better assess the relationships between sleep, fatigue, and depression after intensive care.

It is well‐known that patients with post‐traumatic stress disorder (PTSD) often experience intractable insomnia symptoms.^48^ This study found a significant correlation between PTS symptoms and insomnia severity within a ward setting. PTS symptoms in this setting likely originated during ICU admission.^49^, ^50^, ^51^, ^52^, ^53^, ^54^, ^55^, ^56^ Notably, we found a significant correlation between PTS symptom severity and decreased N3 sleep, which is expected to increase after transfer from the ICU to the ward.^57^ N3 sleep plays a crucial role in memory consolidation and emotional integration, and its deficiency may impair the processing of emotional memories in PTSD patients, leading to increased flashbacks and hyperarousal symptoms.^58^ Furthermore, reduced deep sleep disrupts the hypothalamic‐pituitary‐adrenal (HPA) axis, contributing to chronic neurodegenerative risks and cognitive decline, which could have exacerbated PTS symptoms.^59^

To address these challenges, ICU settings should prioritize alleviating sleep disturbances through practical and patient‐centered approaches tailored to individual needs to promote better sleep quality. Strategies suggested in PADIS guidelines,^60^ including noise and light reduction, structured quiet times, and careful management of sedatives, can help improve sleep architecture.^61^ Additionally, effective pain management using multimodal approaches, such as pharmacotherapy and nerve blocks, alongside psychological support to address stress and emotional distress, can further support recovery.^62^ These interventions aim to improve N3 sleep, potentially contributing to the alleviation of PTS symptoms. Future research should evaluate the effectiveness of ICU sleep strategies in real‐world clinical practice and their long‐term impact on functional recovery, quality of life, and PTS symptom reduction.

This study has several limitations that must be acknowledged. First, the small sample size from a single facility limits the generalizability of our findings. Due to the small sample size, we opted not to conduct multivariate analysis, as this could have led to unstable estimates and overfitting, therefore we were unable to control for potential confounding factors. This includes factors related to the patient's ICU admission and baseline condition, as well as factors already present in our dataset, such as age, gender, type of surgery, and severity of illness, all of which could influence both sleep and other outcomes. Also, despite performing multiple statistical comparisons, we did not apply formal corrections for multiple comparisons, such as the Bonferroni correction or False Discovery Rate correction, as these could have been overly conservative given our exploratory study design and relatively small sample size. Therefore, the possibility of type I errors should be considered when interpreting our results. Second is the potential influence of medication use on EEG results. While the hypnotic medications in this study were mostly limited to melatonin receptor agonists and orexin receptor antagonists (with the exception of one patient who received clonazepam) and their impact on sleep architecture was expected to be minimal,^63^, ^64^ we cannot rule out the possibility that medications, including other pharmaceutical agents, might have affected the EEG findings. Third, the retrospective assessment of preoperative insomnia symptoms may have introduced recall bias. Lastly, the lack of a control group prevents comparisons with patients who did not require ICU admission, making it difficult to ascertain whether the observed sleep patterns and their impact on mental function are specific to ICU patients or a more general phenomenon.

## CONCLUSION

This study explored the relationship between objective and subjective sleep indicators and the physical and mental functioning of patients recovering in the ward after ICU discharge. Our findings confirmed the correlation between insomnia severity and fatigue, depression, and PTS symptoms. Notably, the study found that reduced N3 sleep was associated with PTS symptoms, highlighting the importance of integrated care that emphasizes early detection and treatment of sleep disturbances to mitigate the risk of PTSD progression. Future research should focus on longitudinal studies examining post‐ICU sleep patterns and well‐being after discharge, as well as exploring interventions to improve sleep quality throughout the recovery process.

## AUTHOR CONTRIBUTIONS

Nobuo Sato collected data, performed statistical analyses, and wrote the original draft. Kentaro Matsui supported statistical analysis and edited the manuscript. Masako Arakida collected data. Rie Akaho and Katsuji Nishimura contributed to research design. Takeshi Nomura supervised research design. All authors participated in interpretation of the results and approved the final version.

## CONFLICT OF INTEREST STATEMENT

Katsuji Nishimura is an Editorial Board member of *Psychiatry and Clinical Neurosciences Reports* and a co‐author of this article. To minimize bias, he was excluded from all editorial decision‐making related to the acceptance of this article for publication. The other authors declare no conflicts of interest.

## ETHICS APPROVAL STATEMENT

This study was conducted in accordance with the principles of the Declaration of Helsinki. The study design was approved by the Institutional Review Board of Tokyo Women's Medical University (no. 5690).

## PATIENT CONSENT STATEMENT

Written informed consent was obtained from each respondent.

## CLINICAL TRIAL REGISTRATION

N/A.

## Data Availability

The data of this study are available from the corresponding author upon reasonable request.
